# MiRNA-543 promotes osteosarcoma cell proliferation and glycolysis by partially suppressing PRMT9 and stabilizing HIF-1α protein

**DOI:** 10.18632/oncotarget.13672

**Published:** 2016-11-28

**Authors:** Heng Zhang, Xiaofeng Guo, Xing Feng, Tingting Wang, Zhaohua Hu, Xiangyong Que, Qingsong Tian, Tianbo Zhu, Guixian Guo, Wei Huang, Xinzhi Li

**Affiliations:** ^1^ Department of Orthopedics, Renhe Hospital, China Three Gorges University, Yichang, Hubei, China; ^2^ Department of Molecular Pharmacology, Rutgers University, New Brunswick, New Jersey, USA; ^3^ Department of General Surgery, Beijing Friendship Hospital, Capital Medical University, Beijing, China; ^4^ National Clinical Research Center of Digestive Diseases, Beijing, China; ^5^ Department of Medicine, Medical College, China Three Gorges University, Yichang, Hubei, China; ^6^ Department of Medicine, The Second Hospital Affiliated to Guangzhou Medical University, Guangzhou, Guangdong, China; ^7^ Medical College of Xiamen University, Xiamen, Fujian, China

**Keywords:** osteosarcoma, miRNA-543, glycolysis, PRMT9, HIF-1α

## Abstract

Osteosarcoma (OS) is the most common primary bone tumor, occurring frequently in adolescents and possessing a high malignant severity. MicroRNAs play critical roles during OS development. Thus, elucidation of the involvement of specific microRNAs in the development of OS may provide novel therapeutic targets for OS treatment. Here, we showed that in the OS specimens from patients, the levels of miR-543 were significantly increased whereas the levels of PRMT9 were significantly decreased, compared to the paired normal bone tissue. Moreover, miR-543 and PRMT9 inversely correlated in the OS cell lines. Bioinformatics analyses predicted that miR-543 may target the 3'-UTR of PRMT9 mRNA to inhibit its translation, which was confirmed by luciferase-reporter assay. MiR-543 promoted OS cell proliferation *in vitro* and *in vivo*. Mechanistically, miR-543 inhibited PRMT9-enhanced cell oxidative phosphorylation, while miR-543 depletion promoted PRMT9-increased HIF-1α instability and inhibited glycolysis in OS cells. Clinically, miR-543 expression was negatively correlated with PRMT9 expression in OS tissues. Together, our data provide important evidence for glycolysis in OS development, and suggest that targeting glycolytic pathway through miR-543/PRMT9/HIF-1α axis may represent a potential therapeutic strategy to eradicate OS cells.

## INTRODUCTION

Osteosarcoma (OS) is primary malignant bone sarcoma in children, and frequently arises in the femur (42%), the tibia and the humerus [1]. Though some advances in the treatment of OS, the efficacy of current strategies for the metastatic and recurrent OS is limited. Especially, the etiology and molecular pathogenesis of OS remain unclear. Thus, it is highly desirable to identify novel biomarkers and therapeutic targets to treat and evaluate OS patients.

One of the most important hallmarks of tumor including OS is changed cellular energetics metabolism [2]. Increased aerobic glycolysis, also known as the Warburg effect, plays a particularly key role in OS cells [3]. Several microRNAs (miRNAs) have recently been found to be involved in cancer cell metabolism [4]. However, the action mechanisms of miRNAs in OS cell glycolysis are unclear.

MiRNAs is a type of short non-coding small RNA, which often inhibits the gene expression via binding to the 3ʹ-untranslated region (3ʹ-UTR) of the target gene [5]. It has been shown that miRNAs dysregulation is correlated with OS development [6]. Some miRNAs may act as tumor suppressors or oncogenes, depending on their expression and target mRNAs [7]. For example, miR-543 functions as a tumor suppressor in endometrial cancer while it is oncogene in hepatocellular carcinomas by targeting PAQR3 [8]. However, the role of miR-543 in the metabolism of OS and action mechanisms remain largely unknown.

This study was aimed to elucidate the roles of miR-543 and protein arginine methyltransferase 9 (PRMT9) in OS cell glycolysis. Our data reveal that in OS tissues and cell lines, miR-543 expression was increased significantly, and that the expression of miR-543 promoted the proliferation of OS cells. Mechanistically, miR-543 increased HIF-1α expression via directly targeting the 3ʹ-UTR of PRMT9, which promotes proliferation and glycolysis in OS cells. In OS tissues, the level of PRMT9 expression was inversely correlated with miR-543 expression. These findings indicate that aerobic glycolysis can promote OS cell phenotype.

## RESULTS

### MiR-543 is increased, and PRMT9 is reduced in OS

**Figure 1:**
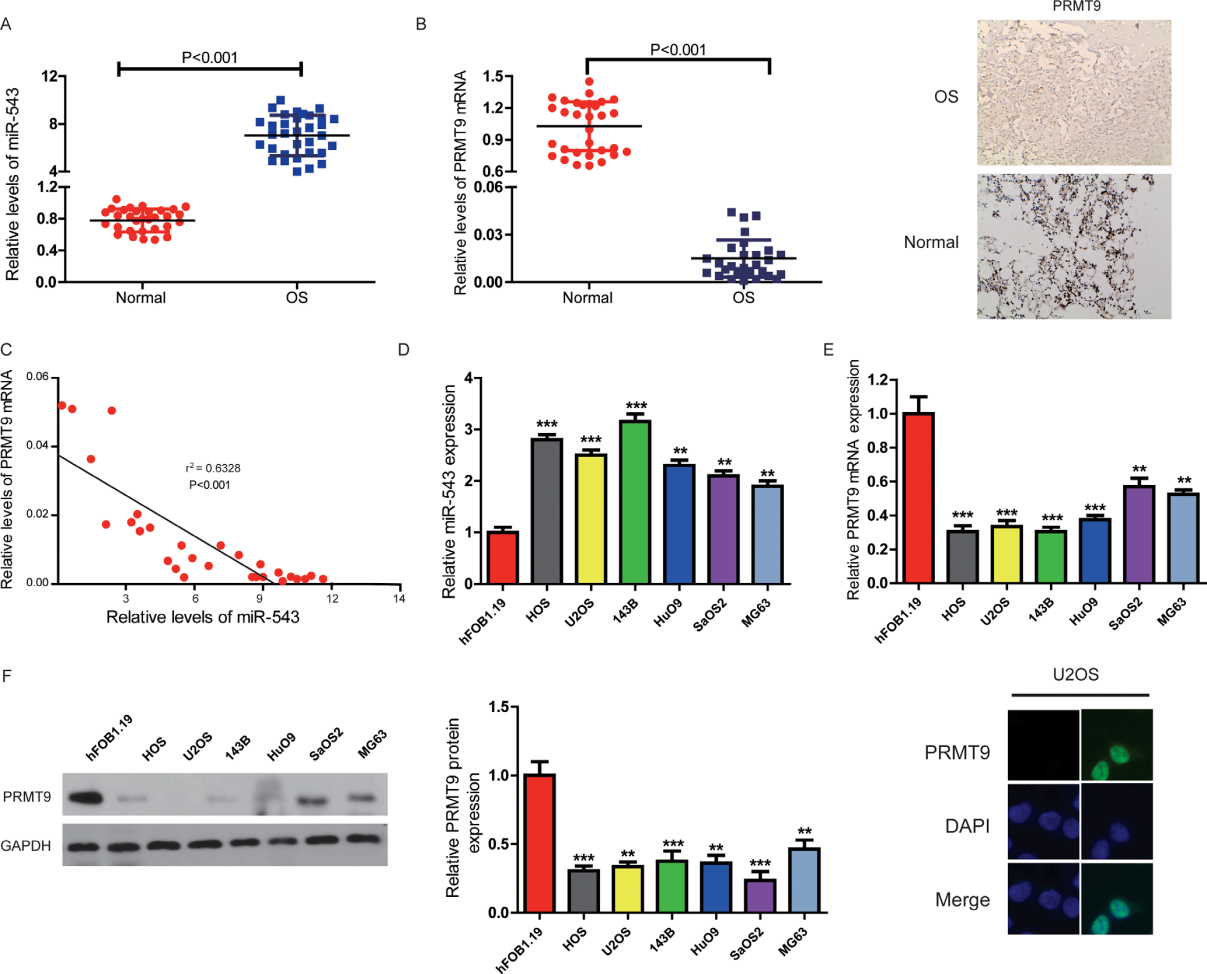
MiR-543 negatively correlated with PRMT9 expression in clinical OS tissues and cell lines (**A** and **B**) Using qRT-PCR, the levels of miR-543 (A) and PRMT9 mRNA (B) in 30 pairs of OS and adjacent noncancerous tissues (such as bone marrow) were quantified. Also, representative PRMT9 immunohistochemistry in OS and adjacent noncancerous tissues (bone marrow as surroundings normal tissues in comparison) were presented (B, right panel). (**C**) A linear regression analysis was used to analyze the correlation of PRMT9 and miR-543 expression in clinical OS samples. (**D**) Compared with that in hFOB 1.19 cells, qRT-PCR shows that miR-543 significantly upregulated in OS cell lines. The expression levels of PRMT9 mRNA (**E**) and protein (**F**) are both markedly decreased in OS cell lines compared with those in hFOB 1.19 cells. The graph (middle-panel) represents densitometric analysis. Moreover, the immunofluorescence pictures of PRMT9 in U2OS cell line were shown (right panel). *P < 0.05, **P < 0.01, ***P < 0.001.

To elucidate the roles of miR-543 and PRMT9, we firstly used qRT-PCR and western blot to assay the expression levels of miR-543 and PRMT9 mRNA in 30 pairs of OS and adjacent normal bone tissues. It was observed that there was a significant increase in the expression of miR-543 in OS tissues compared with their matched normal tissues (Figure 1A), while the expression of PRMT9 was markedly decreased in OS samples (Figure 1B). Furthermore, the statistical analysis of clinical data indicated that miR-543 expression was positively correlated with several OS clinical characteristics including tumor grade, size, and TNM stage, *P <* 0.05 (Table 1), whereas the relationship between miR-543 and PRMT9 was inversely in OS (Figure 1C).

**Table 1:** Relationship between miR-543 and clinical characteristics of osteosarcoma patients

Factor	Characteristic	miRNA-543 expression (*N*)		*P*-values
		Lower	Higher	
**Gender**	Male	23	11	*p* = 0.510
	Female	24	8	
**Age (years)**	< 18	20	7	*p* = 0.669
	> = 8	27	12	
**TNM stage**	I	18	13	*p* = 0.027*
	II~III	29	6	
**Tumor location**	Femur	32	10	*p* = 0.670
	Tibia	4	2	
	Humerus	4	3	
	Others	7	4	
**Metastases**	Lung	23	10	*p* = 0.023*
	Other	4	6	
	No	20	3	
**Recurrence**	Yes	13	12	*p* = 0.007**
	No	34	7	
**Tumor maximum diameter (cm)**		2.14 ± 0.21	3.34 ± 0.24	*P* = 0.002^Δ^

* *p* < 0.05,

** *p* < 0.01, Chi-square test.

Δ *P* < 0.01, student's *t* test.

Next, we analyzed the levels of miR-543 in six OS cell lines and human osteoblastic cell line hFOB 1.19. As indicated in Figure 1D, all of OS cell lines had higher expression of miR-543 than hFOB 1.19 cells, while there was a significant reduction in the levels of PRMT9 in OS cell lines compared with hFOB 1.19 cells (Figure 1E and 1F). Together, our data suggest that miR-543 might promote whereas PRMT9 might inhibit OS development.

### MiR-543 promotes OS cell proliferation

**Figure 2:**
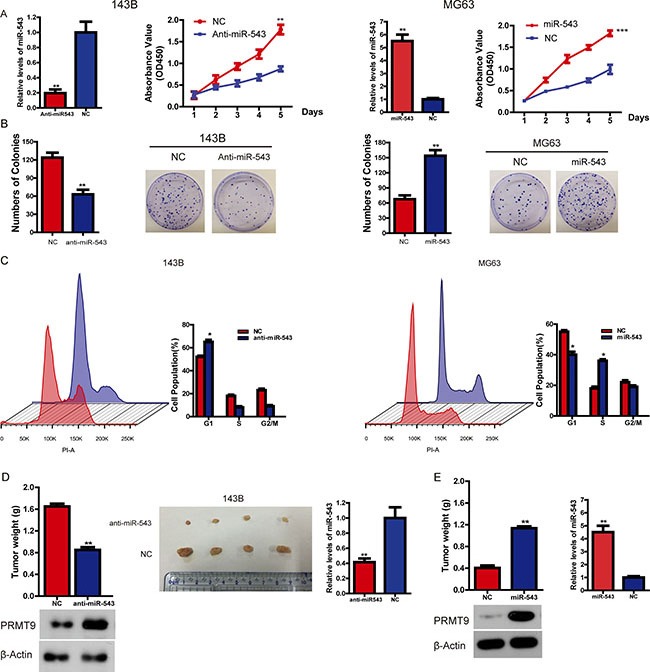
MiR-543 promotes OS cell growth *in vitro* and *in vivo* (**A**) The effect of miR-543 on OS cell growth was analyzed using the CCK-8 assay. (**B**) The role of miR-543 in colony formation in MG63 and 143B cells was analyzed. (**C**) Flow cytometry analysis was used to analyze OS cells in G1, S, and G2/M phase. (**D** and **E**) The role of miR-543 in tumor formation in a mice xenograft model. Nude mice (*n* = 10 per group) were injected subcutaneously into opposite flanks with 1.5 × 10^6^ control cells or cells transfected with anti-miR-543 (D), or miR-543 -overexpressed cells (E). The mice were sacrificed, and the tumors were then removed, weighed and compared. Also, the levels of both PRMT9 and miR-543 were measured via western blot and real-time PCR respectively. The results are presented as means ± SD. Statistical significance was concluded at *P < 0.05, **P < 0.01, ***P < 0.001.

Having observed that the levels of miR-543 are correlated with poor survival in OS patients, we set out to functionally characterize the effects of miR-543 on OS cells. Firstly, OS cell proliferation experiments demonstrated that overexpression of miR-543 greatly enhanced the growth rates of MG63 cells, whereas silencing miR-543 expression significantly inhibited the proliferation of 143B cells (Figure 2A). The pro-proliferation function of miR-543 in OS cells was further confirmed using colony formation assays (Figure 2B). Additionally, the results above described were supported by data from cell-cycle assays. Knockdown of miR-543 was found to lead to *G1*-*S* arrest and a reduction in the proportion of cells in *S* and *G2/M* phase in 143B cells, whereas overexpression of miR-543 in MG63 cells presented the opposite phenotype (Figure 2C). Importantly, an *in vivo* tumor formation assay in a nude mouse model demonstrated that compared with the control, miR-543 overexpression significantly promoted the tumorigenesis of OS cells, while miR-543 knockdown caused the opposite phenotype (Figure 2D and 2E). Together, these data clearly indicate that miR-543 functions as an oncomiR in OS.

### MiR-543 decreases PRMT9 expression by directly binding to its 3ʹ-UTR

**Figure 3:**
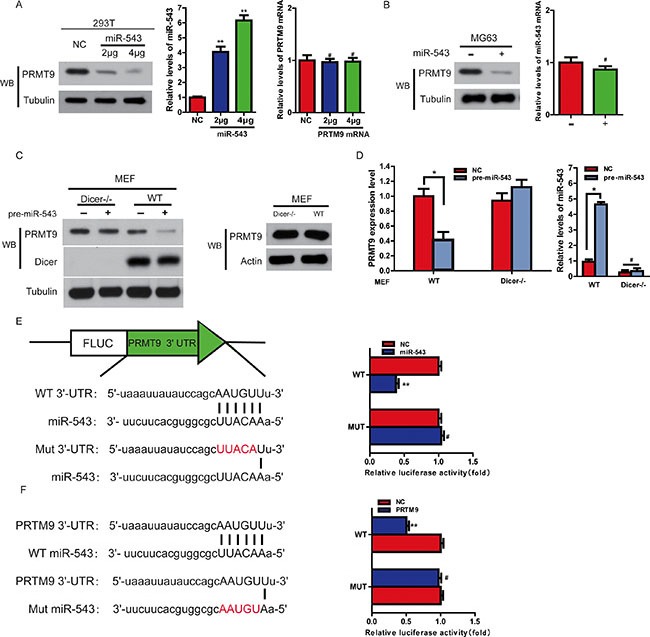
MiR-543 decreases PRMT9 expression by directly binding to its 3ʹ-UTR (**A**) An increasing amount of pre-miR-543 plasmid were transfected into HEK293T cells. PRMT9 expression was analyzed with immunoblotting. (**B**) A similar experiment as in (A) was performed in MG63 cells. (**C** and **D**) MEF WT and MEF Dicer−/− cells were transfected with pre-miR-543 or control vector. Mature miR-543 was measured by quantitative real-time PCR (D). And PRMT9 expression was determined by western blot (C) and quantitative real-time PCR (D). *P < 0.05. (**E**) Schematic representation of the luciferase reporter plasmids containing the PRMT9 3ʹ-UTR and putative wild-type or mutant miR-543-binding sequence in the 3ʹ-UTR of PRMT9 mRNA. HEK293T cells were cotransfected with a control vector or miR-543 and a luciferase reporter construct containing the wild-type or mutant PRMT9 3ʹ-UTR. The data were normalized, and the luciferase activity of the control was set to 1. (**F**) HEK293T cells were cotransfected with a control vector or wild-type or mutant miR-543 which was mutated in binding sequence to the 3ʹ-UTR of PRMT9 mRNA and a luciferase reporter construct containing the wild-type PRMT9 3ʹ-UTR. The data were normalized, and the luciferase activity of the control was set to 1.

To further evaluate the function and action mechanisms of miR-543, it is important to determine a direct target gene of miR-543 in OS tumorigenesis. To this end, we used the miRanda and TargetScan to predict target gene of miR-543. Among the target genes predicted by the miRanda, TargetScan and starBase, PRMT9 attracted our attention because its 3ʹ-UTR contains a putative target sequence for miR-543, and PRMT9 is closely involved in cancer cell proliferation [11]. Indeed, overexpression of miR-543 significantly decreased the level of PRMT9 in both MG63 and HEK293T cells (Figure 3A and 3B). Consistent with the role of Dicer as a central regulator of miRNA mature [12], overexpression of miR-543 greatly reduced the levels of PRMT9 only in wild-type MEF (+/+) cells, not in Dicer-knockout MEF cells (Figure 3C and 3D). Therefore, Dicer is indispensable for the inhibition of PRMT9 by miR-543. Next, we performed a firefly luciferase reporter analysis to confirm that miR-543 directly inhibited PRMT9. As shown in Figure 3E, miR-543 significantly inhibited the reporter activity of PRMT9 with wild-type 3ʹ-UTR, while the activity of PRMT9 with the mutant 3ʹ-UTR construct could not be suppressed. We also created mutant version at ‘5’-seed” region of miR-543, and found that co-expression of wild-type miR-543, not mutant construct, significantly inhibited the expression of the PRMT9 3ʹ-UTR reporter (Figure 3F). These results suggest that PRMT9 3ʹ-UTR can directly be targeted by miR-543 in OS cells.

### MiR-543 enhances OS cell proliferation via glycolysis which can be inhibited by PRMT9

**Figure 4:**
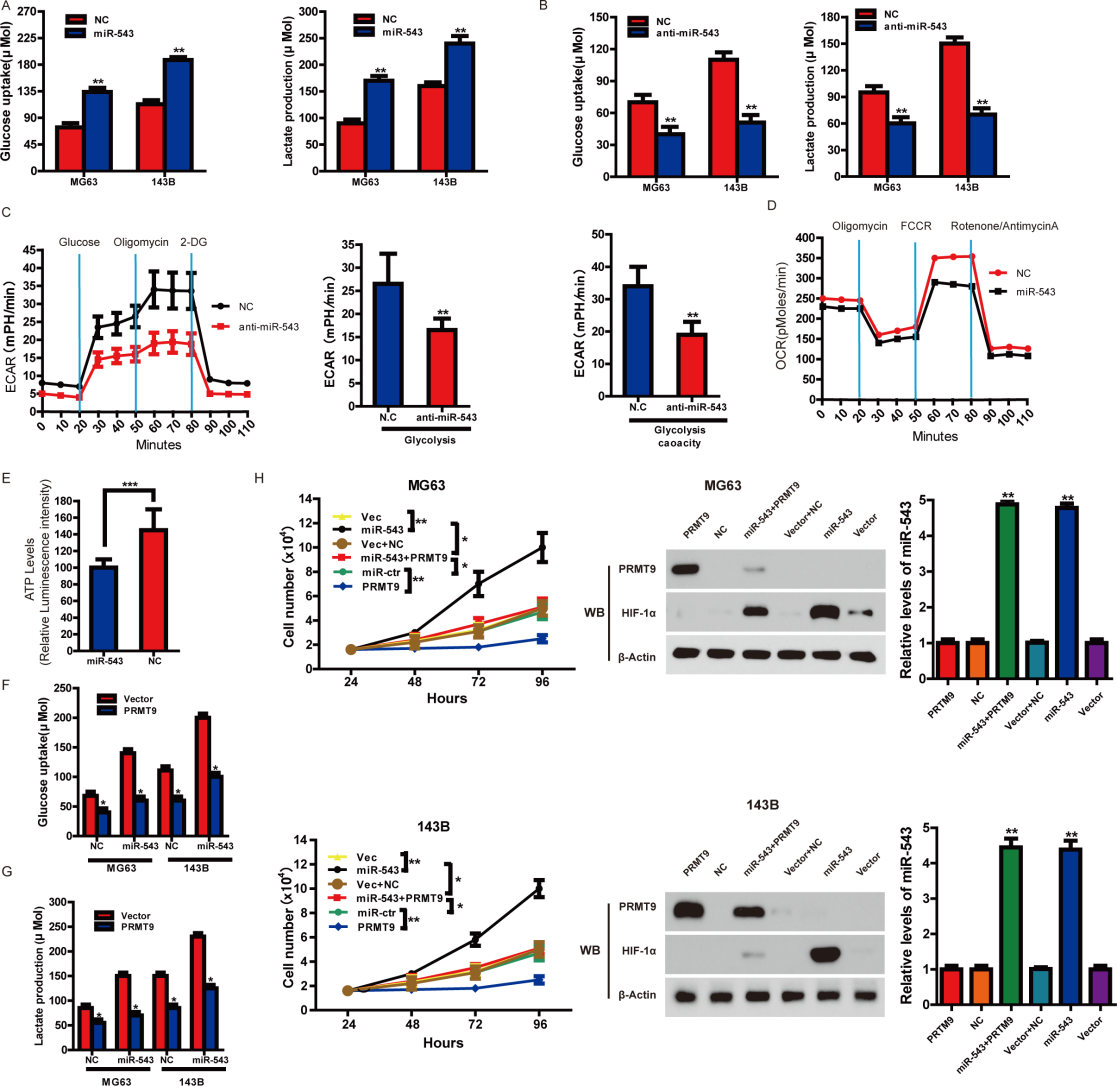
PRMT9 can inhibit miR-543-induced glycolysis and cell proliferation (**A** and **B**) The levels of lactate production and glucose uptake were measured after the transfection with indicated oligonucleotides. (**C**) Real-time measurement of ECAR showed that anti-miR-543 inhibits glycolysis and glycolytic capacity in OS cells. (**D**) Oxygen consumption rate (OCR) in miR-543-expressing and control cells was measured. OCR was measured after sequential incubation with 2 μM oligomycin, 2 μM FCCP and 0.5 μM Rotenone/antimycin A. (**E**) Cellular ATP level was measured by luminescence microplate reader with ATPlite assay kit. Results were normalized to cellular protein concentrations. MG63 and 143B cells were transfected with control vector or PRMT9-expression vector followed by the scrambled oligonucleotide or miR-543 mimics. After transfection, the level of glucose uptake (**F**) and lactate production (**G**) were measured. (**H**) MG63 and 143B cells were transfected with control vector, PRMT9-expression vector, control vector + scrambled oligonucleotide, PRMT9 expressing vector + miR-543 mimics, scrambled oligonucleotide or miR-543 mimics. The number of cells was counted. Moreover, the protein and mRNA levels of miR-543, PRMT9 and HIF1a were measured. *P < 0.05, **P < 0.01.

To further explore the biological effects of miR-543 in OS, we evaluated the differences in metabolic parameters. As shown in Figure 4A, compared with the control group, enhanced expression of miR-543 significantly influenced aerobic glycolysis in OS cells by increasing lactate production and glucose uptake, whereas knockdown of miR-543 indicated an opposite phenotype (Figure 4B). The extracellular acidification rate (ECAR) measurements showed that anti-miR-543 decreased glycolysis and glycolytic capacity in OS cells (the non-glycolytic acidification level was relatively low) (Figure 4C). We also measured oxygen consumption rate (OCR). It was observed that basal and maximal OCRs were all higher in control cells compared to miR-543-expressing cells (Figure 4D). These data suggest that miR-543-expressing cells possess more glycolytic phenotypes and less mitochondrial respiration than control cells. Furthermore, compared to control cells, the intracellular ATP level was lower in miR-543-expressing cells (Figure 4E). These results indicated that miR-543 played vital roles in aerobic glycolysis in OS cells.

The above results prompted us to validate that PRMT9 might inhibit the glycolysis and cell proliferation of OS induced by miR-543. For this purpose, MG63 and 143B cells were transfected with control vector, miR-543 mimics, PRMT9-expressing vector, PRMT9- expressing vector + miR-543 mimics, respectively. The results showed that increased glycolysis induced by miR-543 could be repressed by PRMT9, leading to decreased glucose uptake (Figure 4F) and lactate production (Figure 4G). Next, we explored whether PRMT9 could inhibit miR-543-induced cell proliferation. It was observed that miR-543-promoted cell proliferation was reversed by increased PRMT9 (Figure 4H). These results confirmed that miR-543 promoted glycolysis and cell proliferation through targeting PRMT9.

### Overexpression of miR-543 enhances glycolysis by decreasing PRMT9-induced HIF-1α instability

**Figure 5:**
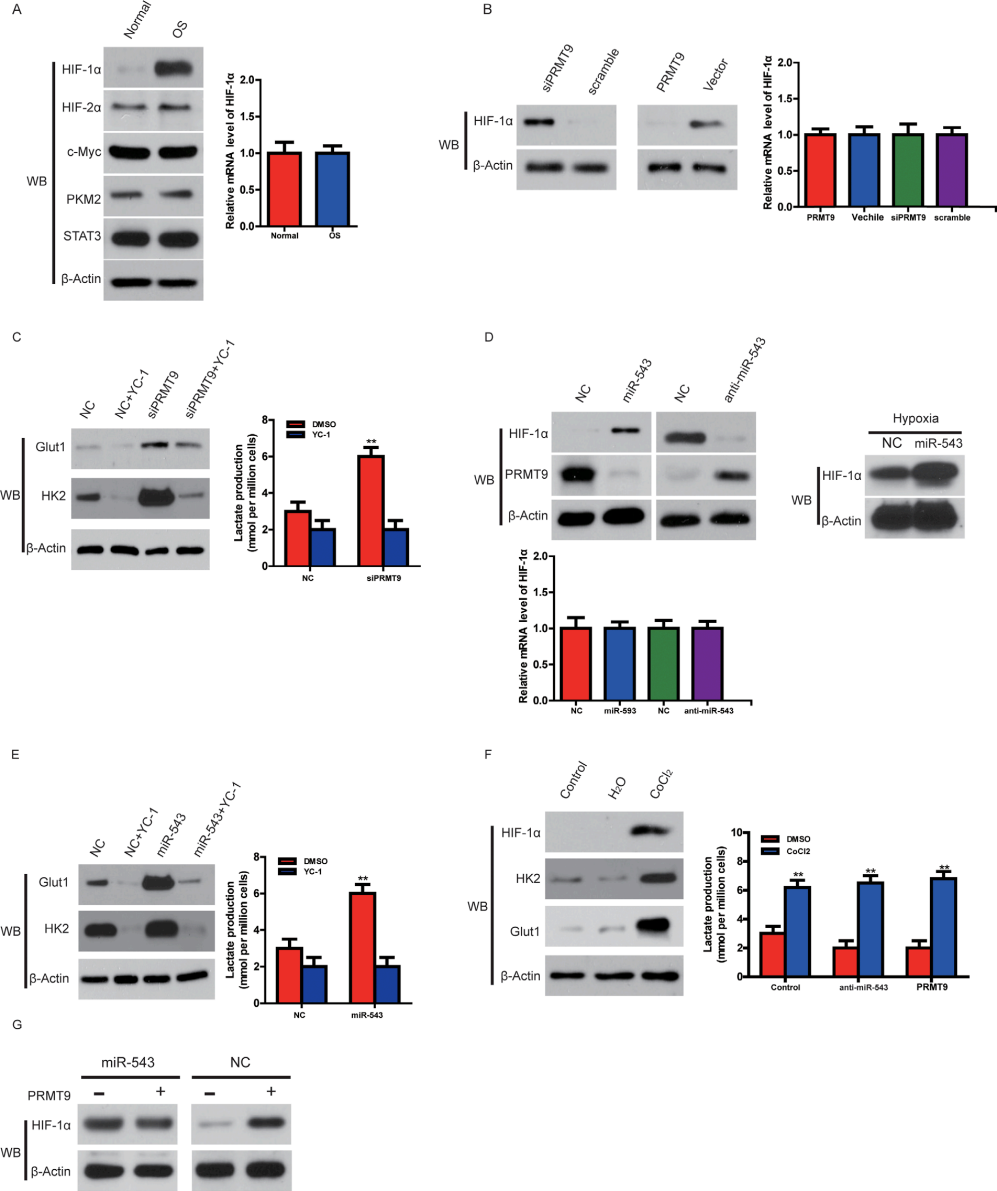
Overexpression of miR-543 enhances glycolysis by decreasing PRMT9-induced HIF-1α instability (**A**) The expression of HIF-1α, HIF-2α, c-Myc in OS and normal control analyzed using Western blot (left panel). The expression levels of HIF-1α mRNA in OS and normal control was assayed with quantitative RT-PCR (right panel). (**B**) The effect of PRMT9 overexpression or knockdown on the protein level of HIF-1α in 143B cells (left panel); the effect of PRMT9 overexpression or knockdown on the level of HIF-1α mRNA in OS cells (right panel). (**C**) The HIF-1α inhibitor YC-1 or knockdown of PRMT9 oppositely regulates expression of HK2 and Glut1 in 143B cells (left panel); lactate production in the same cells (right panel). (**D**) The protein level (left panel) or mRNA level (right panel) of HIF-1α with overexpression or knockdown of miR-543 under normoxia and hypoxia condition. (**E**) The expression of HK2 and Glut1 in 143B cells treated with YC-1 and/or miR-543 overexpression (left panel); lactate production in OS (right panel). (**F**) CoCl_2_ affected on the expression of HK2 and Glut1 (left panel), and lactate production (right panel) in 143B cells with PRMT9 overexpression or miR-543 knockdown. (**G**) The effect of PRMT9 on the protein level of HIF-1α in 143B cells with or without miR-543 overexpression. Also, PRMT9 expression was determined by western blot.

Given that HIF-1α is one of key transcription factors in regulating glycolysis [13] and PRMT9 decreases the stability of HIF-1α protein, we hypothesized that HIF-1α may decide the miR-543 -induced glycolysis in OS cells. To evaluate this idea, we firstly measured the level of HIF-1α in OS. As shown in Figure 5A, the level of HIF-1α protein was greatly enhanced in patients’ samples, with no significant changes for c-myc or HIF-2α or the levels of HIF-1α mRNA in normal control and OS tissues, suggesting that high HIF-1α levels in OS likely result from enhanced protein stability rather than increased transcription. Furthermore, overexpression of PRMT9 decreased HIF-1α, whereas its knockdown increased HIF-1α, with HIF-1α mRNA levels obviously unchanged by both treatments (Figure 5B).

Then we set out to demonstrate whether HIF-1α is responsible for PRMT9 knockdown-enhanced glycolysis, OS cells were treated with the HIF-1α inhibitor YC-1 [14]. Although PRMT9 knockdown was found to increase the levels of Glut1 and HK2, two HIF-1α ʹs target genes, and lactate production in OS cells, YC-1 treatment reversed these increases (Figure 5C). Furthermore, miR-543 knockdown decreased, whereas overexpression of miR-543 enhanced HIF-1α protein, but not mRNA, under normoxia and hypoxia condition (Figure 5D). MiR-543 overexpression was found to not only enhanced lactate production, but also the levels of HK2 and Glut1; however, YC-1 inhibited the miR-543-induced lactate production and Glut1 and HK2 expression (Figure 5E).

Consistent with these data, CoCl_2_, which has been shown to be a chemical inducer of HIF-1α protein [15], enhanced the expression of HK2 and Glut1 and lactate production in OS cells. However, neither miR-543 knockdown nor PRMT9 overexpression blocked the HIF-1α or CoCl_2_ -induced increase of HK2 and Glut1 expression and lactate production (Figure 5F). Furthermore, we found that HIF-1α knockdown could inhibit cell proliferation induced by miR-543 (Supplementary Figure S1A and S1B). Additionally, miR-543 upregulation cannot increase HIF1a protein level after knocking down PRMT9 in OS cells (Figure 5G), suggesting that miR-543 induced HIF1a stabilization is dependent on PRMT9. Collectively, these data indicate that HIF-1α is responsible for the changes in glycolysis induced by miR-543 overexpression or PRMT9 downregulation. In addition, miR-543-induced HIF-1α could promote endothelial cells proliferation and migration (Supplementary Figure S2A and S2B).

### Clinical significance and relevance of miR-543, PRMT9 and HIF-1α expression in patients with OS

**Figure 6:**
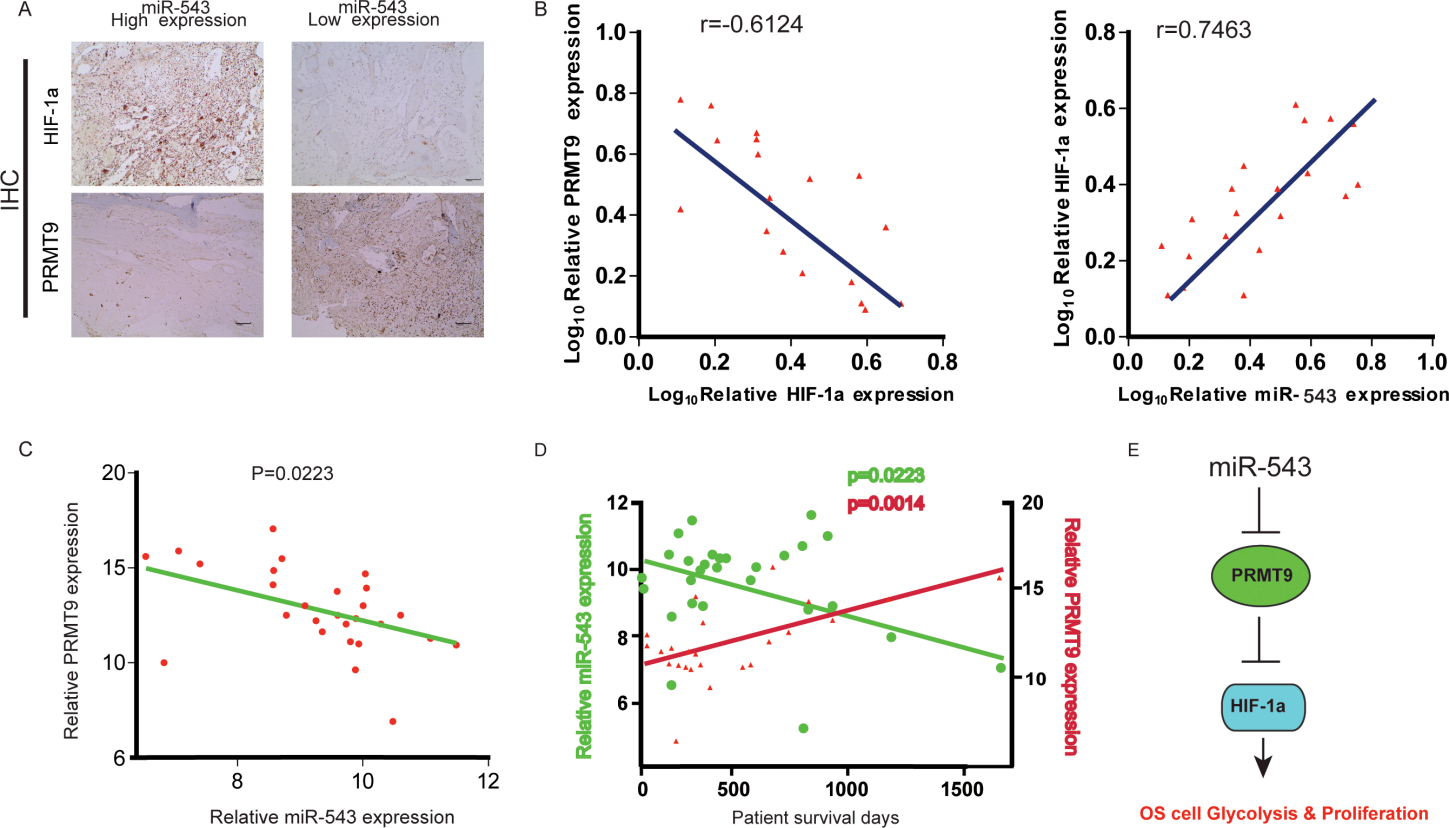
Clinical significance and correlation of miR-543, PRMT9 and HIF-1α expression in patients with OS (**A**) Representative images of IHC staining for PRMT9 and HIF-1α in low and high miR-543 expression OS cases. (**B**) Correlation between miR-543 expression and PRMT9 and HIF-1α expression in the OS samples. (**C** and **D**) RNA was extracted from 36 OS patient biopsies, representing long-term (> 2 yr) and short-term (< 1 yr) survival after diagnosis. The expression of miR-543 and PRMT9 was determined by qPCR (*n* = 3), and normalized to the expression of U6A and actin, respectively. (**E**) Schematic diagram of the regulatory pathway from miR-543 to glycolysis.

Given that miR-543 downregulates PRMT9 and upregulates HIF-1α expression in OS cells, we wondered whether this pathway also functions *in vivo*. We examined miR-543 expression and its correlation with PRMT9 and HIF-1α in clinical OS tissues. To this end, miR-543 expression was evaluated using *in situ* hybridization (ISH) in a cohort of 36 OS samples, followed by immunohistochemistry (IHC) staining for PRMT9 and HIF-1α. An inverse expression pattern between PRMT9 and miR-543 or HIF-1α was consistently observed, whereas a positive expression pattern between miR-543 and HIF-1α was observed (Figure 6A–6C). Moreover, there was a statistically significant linear relationship between PRMT9 expression and patient survival in OS, while there was an inverse relationship between miR-543 expression and OS patient survival (Figure 6D). Taken together, these results showed that PRMT9 is a positive predictor of OS patient survival. These data confirm the hypothesis that miR-543 is an oncogene, while PRMT9 functions as a tumor suppressor. These clinical data further support mechanism postulating that miR-543 inhibits the expression of PRMT9 that directly suppress HIF-1α to maintain increased glycolysis in OS cells (Figure 6E).

## DISCUSSION

OS are aggressive primary tumor of the bone. Therefore, it is very emergent to explore the molecular mechanisms governing rapidly growth of OS. For the first time, we demonstrated that human OS cells and tissues have higher expression of miR-543 compared with normal controls, and that high level of miR-543 is correlated with advanced clinical stage and high degree of malignancy in OS, which was illustrated by the fact that miR-543 promoted the cell growth and survival capacity of OS cells. We showed that miR-543 exerted oncogenic function on OS via activating HIF-1α signaling. Furthermore, we showed that miR-543 increased the level of HIF-1α by directly targeting the 3ʹ-UTR of PRMT9 mRNA, which promotes OS cell glycolysis and proliferation. More importantly, both miR-543 and HIF-1α expression levels were inversely correlated with the level of PRMT9 in OS samples. This study provided important evidence for glycolysis in OS development, and suggests that targeting glycolytic pathway through miR-543/PRMT9/ HIF-1α axis may represent a potential therapeutic strategy to eradicate OS cells.

Due to the high energy demand but low ATP-generating efficiency in cancer cells, abnormal metabolic phenotypes of cancer cell, such as aerobic glycolysis, is a common feature of cancer [16], and have been considered as potential therapeutic targets for cancer treatments [17]. In this study, for the first time, we explored not only the critical roles of miR-543 in OS aerobic glycolysis and cell proliferation, but also the mechanisms of how miR-543 increased the protein levels and activity of HIF-1α. Our data also confirmed that miR-543 directly targeted PRMT9. PRMT9 upregulation reverted not only miR-543-increased lactate level and glucose uptake, but also miR-543-promoted OS cell proliferation. In turn, metabolic stress has been shown increasing the expression level of miR-543 [18, 19]. Therefore, further research is needed to determine whether there exists a feedback loop between miR-543 and glycolysis. Increasing evidence demonstrated that miRNAs were involved in cancer growth [20]. In particular, miR-543 could function as an oncogene or important tumor inhibitor [8]. By targeting sirt1, miR-543 promoted the progression of gastric cancer [21], whereas in endometrial cancer, miR-543 acts as a tumor suppressor by decreasing the levels of FAK and TWIST1 [22]. Our data clearly indicate that in OS, miR-543 serves as an oncogene. Given the important roles of miR-543 in cancers, the development of miR-543-based gene therapy is encouraged for multiple types of cancer.

Another important aspect of the current study relates to the novel role of the OS-specific PRMT9 in regulation of glycolytic phenotypes and OS growth. PRMT9 not only is a member of the F-box protein family, but also catalyzes protein arginine methylation [23]. Recent study reported that PRMT9 inhibited the stability of HIF-1α, BCL6 and SNAIL [24, 25, 26]. In the present study, downregulation of HIF-1α-targeted genes (Glut1 and HK2) with no change of HIF-1α mRNA was observed following overexpression of PRMT9 under normoxic or hypoxic condition in OS cells, which was similar to a previous study [23]. Moreover, we found that miR-543 does not affect other critical glycolysis-related proteins (such as PKM2, STAT3, Myc) [12]. However, it is not clear whether PRMT9 exerts its effect on HIF-1α in OS cells via the methyltransferase-dependent or the E3 ligase-dependent function. Further investigation is wanted to unveil the deeper mechanism between PRMT9 and HIF-1α pathway in OS.

In summary, our study detected a high expression level of miR-543 in OS, which was associated with poor clinical outcomes. These findings may be due to the fact that miR-543 promoted glycolysis and cell proliferation in OS. We also verified that miR-543 directly targeted PRMT9 in OS, and that its function in glycolysis and cell proliferation could be inhibited by HIF-1α stability. Collectively, these findings imply that the crosstalk between HIF-1α and miR-543 preserved a sustained effect on HIF-1α stabilization, thus making OS cells adaption to hypoxic environments. Therefore, this study sheds new lights on the function of miR-543 in OS, and targeting PRMT9/HIF-1α axis may be a novel, targeted therapeutic strategy for OS.

## MATERIALS AND METHODS

### Measurement of lactate and ATP

According to the manufacturer's protocol, lactate assay kit (Perkin-Elmer Life Sciences) was used to measure the level of lactate. Specifically, the cell culture supernatant was collected, deproteinized with a 10 kDa MWCO spin filter, then diluted with lactate assay buffer, and incubated with lactate probe and lactate enzyme mix at room temperature for 30 min, then measured for absorbance at 570 nm. According to the manufacturer's protocol, cellular ATP contents were measured by using an ATPlite assay kit (Perkin-Elmer Life Sciences). Briefly, 100 μl of cell lysate and substrate solution were mixed. Then the plates were vortexed for 2 min at 700 rpm and incubated for 10 min. Luminescence was assayed with a luminescence analyzer (Berthold LB960).

### Mouse models

All animal experiments were approved by the Animal Ethics Committee of University. Four-week-old male nude mice (laboratory animal center of Shanghai, China) were housed under pathogen-free condition. Two millions OS cells (at the concentration of 2 × 10^7^ cells/ml) with overexpression of indicated plasmids were mixed with 50% Matrigel and subcutaneously injected into nude mice. All animal experiments were approved by the Animal Ethics Committee of University. Four-week-old male nude mice (laboratory animal center of Shanghai, China) were housed under pathogen-free condition. Two millions OS cells with overexpression of indicated plasmids were mixed with 50% Matrigel and subcutaneously injected into nude mice. We calculated Tumor size using ½ a2b (a represented the short axis, whereas b represented the long axis of tumor). The growth curve was plotted based on the mean tumor volume ± SEM. Thirty days after injection, the animals were sacrificed, and tumors were harvested, measured, weighed and fixed in 10% formalin. Tumor weight was calculated as mean ± SD from the animal in each group. Protein lysate was prepared from the tumors for western blot analysis. Protein lysate from the tumors was prepared for western blot analysis.

### Cell culture

Human fetal osteoblastic cell line (hFOB) and OS cell lines (Saos2, MNNG/HOS, U2OS, and MG63) and HEK293T were purchased from the Institute of Cell Bank for Biological Sciences (Shanghai, China). Human osteosarcoma 143B cells (ATCC CRL-8303), suitable transfection host cells, are thymidine kinase deficient (TK-) and resistant to BUdR [9]. Cells were maintained at 37°C in a humidified air atmosphere containing 95% air and 5% CO2 in DMEM or RPIM-1640 with 10% fetal bovine serum (Invitrogen), 100U/ml penicillin (Sigma-Aldrich) and 100mg/ml streptomycin (Sigma-Aldrich). HFOB was cultured according to ATCC protocols. MEFs were prepared and cultured in DMEM containing 10% fetal bovine serum (FBS), 2 mM L-glutamine, 1× nonessential amino acids and 0.1 mM 2-mercaptoethanol (Invitrogen) as described before. To remove functional Dicer, MEFs were treated with Adreno-Cre virus, added at a multiplicity of infection (MOI) of ~100 and performed further analysis.

### OS patients tissues

All patients agreed to participate in the study and gave informed consent. The study was approved by the Ethics Committee of University. The 66 paired samples of human OS and their matched adjacent non-cancerous tissues were collected at the time of surgery between 2013 and 2015 at the Department of Orthopedics, Huazhong University of Science & Technology Affiliated Tongji Hospital (Wuhan, China). Human OS specimens were immediately frozen in liquid nitrogen and stored at –80°C in the refrigerator.

### Cell proliferation and cell cycle assay

CCK8 assay (Dojindo, Japan) was adopted to evaluate cell growth. After transfection, 3000 cells in 100 ul complete medium were seeded into the 96-well plate. After incubation 24, 48, 72, 96, and 120 h, 10 ul CCK8 assay solution was added to each well. Then, after incubation another 2 h, optical density (OD) at 450 nm was measured with Enzyme immunoassay analyzer (Thermo Fisher Scientific, Inc., USA). Each sample was measured in triplicate. For cell cycle assay, the cells were fixed in 70% ethanol at –20°C 48 hours after transfection for 12–24 hours. After that, cells were treated with a staining solution, which containing 50 ug/ml propidium iodide (PI) (Bio-Rad, USA) and 50 ug/ml RNase A (Bio-Rad, USA). Each experiment was repeated three times.

### Measurement of oxygen consumption rate and extracellular acidification

OCR was measured as described previously [10]. Briefly, oligomycin (2 μM), FCCP (2 μM), and Rotenone/antimycin A (0.5 μM) were sequentially added. Then the cells in each well were trypsinized and counted; the Seahorse data were normalized to relative cell numbers of each well. XF24 Extracellular Flux Analyzer (Seahorse Bioscience) was used to measure Extracellular Acidification Rate (ECAR). Briefly, the cells (3.5 × 10^4^ per well) were plated in the XF24 microplate and cultured with XF assay medium. Glucose, oligomycin (2.5 μM), and 2-deoxyglucose (100 mM) were sequentially used in an XF24 flux analyzer.

### RNA isolation and reverse transcription-quantitative polymerase chain reaction (RT-qPCR)

Total RNA and miRNA were isolated using the RNeasy Mini and miRNeasy Mini kits (Qiagen, Inc.). Reverse transcription and RT-qPCR were set up to create a catalytically inactive PRMT9 mutant containing four mutations (L182A, D183A, I184A, and G185A). Mutant primers were as follows:

rmed using Applied Biosystems TaqMan miRNA assay kits (Thermo Fisher Scientific, USA) and Applied Biosystems hsa-miR-26a (cat. no. 4395166; Thermo Fisher Scientific). The Bio-Rad iCycler IQ RealTime PCR Detection System (Bio-Rad Laboratories) was used. The following primers (Sangon Biotech Co., China): Forward, 5ʹ- CCAGCTACACTGGGCAGCAGCAAT TCATGTTT-3ʹ and reverse, 5ʹ-CTCAACTGGTGTCGT GGA-3ʹ for miR-543; forward, 5ʹ-CTCCCTCCCGAATT TGAAG-3ʹ and reverse, 5ʹ-GTCAGAGGGAGAGG TCAG-3ʹ for PRMT9; and forward, 5ʹ-GCGCGTCGTG AAGCGTTC-3ʹ and reverse, 5ʹ-GTGCAGGGTCCGAG GT-3ʹ for U6. The RT-qPCR data were normalized using the 2^−ΔΔCq^ method, relative to glyceraldehyde-3-phosphate dehydrogenase or U6 small nuclear RNA.

### Measurement of glucose consumption

The control or LDHA-expressing vector was transfected into OS cell lines. Cell culture media were collected 48 hours after the transfection. Glucose uptake was measured using Amplex Red Glucose/Glucose Oxidase Assay Kit (Invitrogen, USA). Total cellular protein amounts normalized the results.

### Colony formation assay

In total, 500 transfected OS cells were plated into a 6-well plate and cultured in DMEM supplemented with 10% FBS for two weeks. Then the cells were fixed and stained with methanol for 20 min, followed by 0.5% crystal violet for 15 min. The colonies were quantified using an inverted microscope (IX83; Olympus Corporation, Japan).

### Western blot analysis

Western blots were analyzed using ImageJ 1.48u software (National Institutes of Health, USA). The protein was collected from the OS or paired non-tumor tissue from the OS patient, or from cultured cells, in RIPA buffer (100 μg/ml phenylmethylsulfonyl fluoride, 1% NP40, 0.1% SDS, 0.5% sodium deoxycholate, in PBS) on the ice. The supernatants were collected after centrifugation at 12000 × g at 4°C for 20 min. Protein concentration was determined using a BCA protein assay kit (Bio-rad), and whole lysates were mixed with 4×SDS loading buffer (125 mmol/l Tris-HCl, 4% SDS, 20% glycerol, 100 mmol/l DTT, and 0.2% bromophenol blue) at a ratio of 1:3. Samples were heated at 100°C for 5 min and were separated on SDS-polyacrylamide gels, transferred to a PVDF, and probed with a primary antibody. After incubation with horseradish peroxidase-conjugated second antibody, autoradiograms were prepared using the enhanced chemiluminescent system to visualize the protein antigen. The signals were recorded using X-ray film. Primary antibodies were rabbit anti-PRMT9, anti-HIF-1α, anti-β-Actin (Sigma-Aldrich) and anti-α-tubulin (Cell Signaling, USA). The secondary antibody is HRP-conjugated anti-rabbit (Jackson Immuno Research Labs, USA). Blotting images were representative from 5 repeats. We used α-tubulin as a protein loading control.

### Cell transfection and plasmids and antibodies

The anti-Hif-1α antiserum was generated in rabbits using a bacterially expressed fragment containing amino acids 418–698 of human HIF-1α, and anti-PRMT9 was purchased from Novus (CO, USA). Anti-Flag was obtained from Sigma–Aldrich. Recombinant lentivirus vectors carrying the negative control miRNA (NC), miR-543 precursor (miR-543) or miR-543 inhibitor (anti-miR-543) (GenePharma, China) were transduced into the cells according to the manufacturer's protocol. Cells were then cultured in the Polybrene supplemented medium (Santa Cruz Biotechnology, USA) after incubation in a normal medium for 24 h. Then, the lentivirus infected cells were cultured in 12-well plates overnight. After that, the medium was replaced with normal medium. Subsequently, the stable clones expressing the miR-543 or anti-miR-543 were selected using puromycin (Santa Cruz Biotechnology). A pEGFP-C1 vector (Clontech), a pGEX-6p-1 vector (GE Healthcare Life Sciences), a pEGFP-C1 vector (Clontech) and a 3XFlag vector (Invitrogen) were used to subclone human PRMT9 cDNA (NM_138364.2). The sequence of the siRNA targeting PRMT9 was 5ʹ-UUGAGCAAUUCACGUUCAUTT-3ʹ, corresponding to nucleotides 620–644 in its coding region. The sequences of siRNAs targeting PRMT9 were 5ʹ-UAGUCCAUACCAACUUCGUAGAAAA-3ʹ (siPRMT9), corresponding to nucleotides 150–174 in its coding region.

### Immunohistochemical assays

Immunohistochemical staining was carried out to assess the protein expression pattern of PRMT9 and HIF-1α in paraffin-embedded human OS tissues. Briefly, the paraffin-embedded tissue blocks from OS patients were cut into 4-μm-thick sections and baked at 65° for 30 min. Then the sections were deparaffinized with xylene, followed by rehydrated in the water, submerged into EDTA antigenic retrieval buffer and microwaving was processed for antigen retrieval. Next, endogenous peroxidase activity was blocked by 3% hydrogen peroxide for 15 min, followed by incubation with 1% bovine serum albumin to block the nonspecific binding. The specimens were incubated overnight at 4°C with a rabbit polyclonal antibody against indicated antibodies. The antibody was replaced with normal goat serum as negative controls. After washing with PBST, the tissue slides were incubated with a biotinylated anti-rabbit secondary antibody (Santa Cruz) at room temperature for 30 min, followed by further incubation with streptavidin-horseradish peroxidase complex (Santa Cruz) at room temperature for 30 min. The slides were counterstained with 10% Mayer's hematoxylin and mounted in Crystal Mount.

### Luciferase reporter activity assay

Luciferase-reporters were successfully constructed using molecular cloning technology. The genomic DNA of human 293T cells was used to amplify the 3-UTR of PRMT9 by PCR; then the PCR product was purified and cloned into pcDNA3.1-luc, resulting in the wild-type PRMT9 reporter plasmid. The mutant PRMT9 reporter plasmid was constructed by mutating the binding sites of miR-543 in the 3ʹ-UTR of PRMT9 using PCR-based site-directed mutagenesis according to manufacturer's instructions (Stratagene). The primers for amplifying the wild-type 3ʹ-UTR are 5ʹ-GATACTCGA GAGTGAAGAAAATAAGCTGCAATTTTGTACAGAT ACC-3ʹ and 5ʹ-GCGGATCCTCCTAGGAAGTCTCA TTAAAACACT-3ʹ, and the primers for mutant construct are 5ʹ-GATACTCGAGAGTGAAGAAAGAATTCTGCAA TTTTGTACAGATACCAACT-3ʹ and 5ʹ-GCGGATCCT CCTAGGAAGTCTCATTAAAACACT-3ʹ. Reportergene binding assays were performed by co-transfection of 293T cells using wild-type and mutant reporter plasmids pcDNA3.1-Luc-wtUTR and pcDNA3.1-Luc-muUTR with miR-543 overexpressing (or knockdown) plasmid, respectively. pSV40-*Renilla* plasmid was co-transfected as an internal control. Alternatively, reporter gene binding assays were performed by co-transfection of 293T cells using wild-type reporter plasmids pcDNA3.1-Luc-wtUTR with wild-type or mutant miR-543. 24 h later after transfection using the Dual luciferase reporter gene kit (Promega), the ratio of luciferase and Renilla activities was determined. MiR-543-modified cells were seeded in 24-well plates for 24 hours, after which the cells were transfected with 1 μg of Luciferase-reporter plasmids per well. Luciferase activities were measured using the dual-luciferase reporter gene assay kit (Promega, China), according to the manufacturer's instructions.

### *In situ* hybridization (ISH) analysis

*In situ* hybridization procedures were carried out as follows. MiR-543 miRCURYTM LNA custom detection probes (Exiqon, Denmark) were used to perform ISH. The 5ʹ-3ʹ sequences were GCCATCATTACAATGCAGTATCG with a DIG label at both the 5ʹ and 3ʹ ends. Hybridization, washing, and scanning were carried out according to the manufacturer's instructions. The intensities of miR-543 staining was scored and divided into the weak staining (score 0–2), medium staining (score 2–3), and strong staining (score 3–4). The expression scores of greater or equal to 2 was defined as the high expression, less than 2 was the low expression. Patients were stratified into two groups: high miR-543 and low miR-543.

### Statistical analysis

Data were imaged with GraphPad Prism 5 software (Graphpad Software, Inc, USA). All statistical analyses were performed using the SPSS 17.0 statistical software package. A linear regression analysis was used, and bivariate correlations were calculated by Spearman's Rank Correlation Coefficients. All values were depicted as the mean ± standard deviation. Statistical significance was concluded at *P < 0.05, **P < 0.01, ***P < 0.001; #represents no statistical significance. One-way ANOVA was used to analyze statistically all data with a Bonferroni correction, and Fisher's Exact Test was used for comparison of two groups.

## SUPPLEMENTARY MATERIALS AND METHODS


