# Supplementary material for: MiRNA-543 promotes osteosarcoma cell proliferation and glycolysis by partially suppressing PRMT9 and stabilizing HIF-1α protein
Source: Oncotarget. 2016 Nov 28;8(2):2342–55. doi: 10.18632/oncotarget.13672 (PMC5356804; doi:10.18632/oncotarget.13672)
Supplement: Supplementary file 1 [file oncotarget-08-2342-s001.pdf]

MiRNA-543 promotes osteosarcoma cell proliferation and glycolysis by partially suppressing PRMT9 and stabilizing HIF-1α protein

Supplementary Materials

MATERIALS AND METHODS

Migration and invasion assay

Endothelial cell migration was determined by wound-healing assay. Endothelial cells were seeded at a concentration of 3×10^5 cells/ml into the plates with a culture insert (Inbidi, Madison, Wisconsin) to form a gap of 500 μm according to the manufacturer's instruction. After removing the insert, cells that migrated into the wound area were photographed by inverted microscope. The wound closure width is calculated as following: (12– to 72-hour width/0–hourwidth)*100%.

Matrigel Transwells (BD Biosciences, NJ) were used for *in vitro* invasion assay. Briefly, the insert standing in 24-well culture plate were coated with 50 ul Matrigel in serum-free DMEM medium and dried overnight. 1×10^5 endothelial cells in 100 μl serum-free medium were added to the insert. The lower chamber was added 500 μl DMEM medium containing 10% FBS. Cells were incubated for 36 hours and the cells that penetrated the membrane were counted after fixing with the 4% paraformaldehyde and staining with 1% crystal violet.

MTT assay

Endothelial cell proliferation was assessed by 3-(4, 5-dimethyl-2-thiazolyl)-2, 5-diphenyl-2-H-tetrazolium bromide (MTT) assay. Cells were seeded into 96-well plates at 1×10^3/well. For cell proliferation assay, 0.5 mg/ml MTT solution was added to each well for four hours and measured at the absorbance of 570 nm by multifunctional micro plate reader.

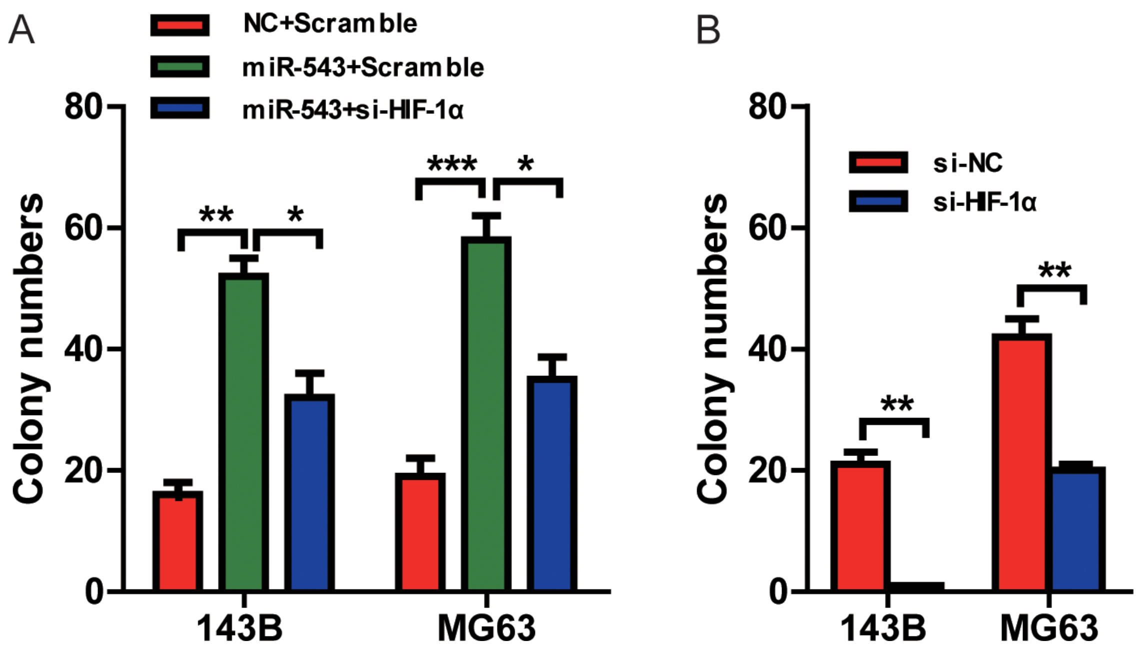

Supplementary Figure S1: The miR-543/PRMT9/ HIF-1α axis regulates the proliferation of OS cells. (**A**) Colony formation assay in OS cells with HIF-1α knockdown. (**B**) HIF-1α knockdown abolished the effects of miR-543 on colony formation in OS cells. *$P < 0.05$, **$P < 0.01$, ***$P < 0.001$. (NC: negative control).

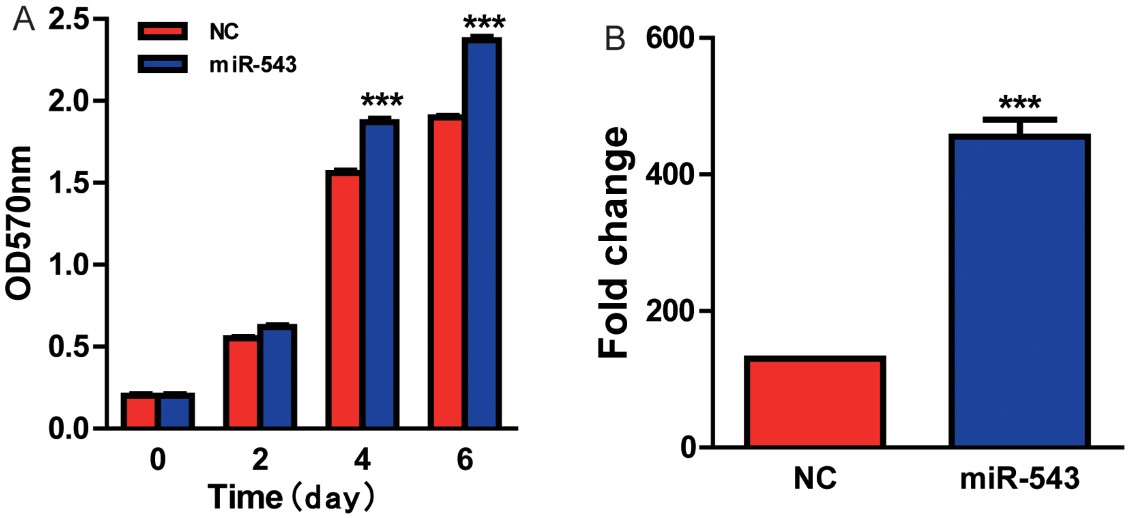

Supplementary Figure S2: Overexpression of miR-543 increases endothelial cell proliferation and migration. (**A**) The effect of miR-543 overexpression on endothelial cells proliferation was determined by MTT analysis. (**B**) Endothelial cells migration was determined by Matrigel transwell assays. Cells at the lower side of the membrane were photographed and counted. The graphic represent the mean and S.D. from three experiments and one way ANOVA was used to determine the statistical significance between miR-543 overexpression and control cells. *** indicates $p < 0.001$. Results are shown as the mean ± SD of three independent experiments.
